# Epicardial left ventricular myocardial rotation correlates with resting myocardial blood flow in type 2 diabetes mellitus patients with angiographically normal coronary arteries

**DOI:** 10.1186/1532-429X-15-S1-E69

**Published:** 2013-01-30

**Authors:** Abdulghani M Larghat, John D Biglands, Ananth Kidambi, John P Greenwood, Sven Plein

**Affiliations:** 1 Multidisciplinary Cardiovascular Research Centre & Leeds Institute of Genetics, Health and Therapeutics, University of Leeds, Leeds, UK; 2 Department Medical Physics, University of Leeds, Leeds, UK

## Background

Type 2 Diabetes Mellitus (T2DM) and prediabetes are associated with an increased cardiovascular risk and heart failure independent of the presence of obstructive coronary artery disease, caused in part by myocardial microvascular dysfunction. Recent studies have reported contractile dysfunction and increased rotation with the onset of diabetic cardiomyopathy in uncomplicated T2DM patients. Independently, higher resting myocardial blood flow (MBF) has been reported in T2DM. The objective of this study was to determine whether there is a correlation between LV Epicardial Rotation (LV ER) and resting MBF in T2DM patients with angiographically normal coronaries, measured by tissue tagging and perfusion cardiovascular Magnetic Resonance (CMR).

## Methods

Sixty five patients with no coronary stenosis >30% on invasive angiography were recruited and categorized into T2DM, prediabetes and normal controls groups according to American Diabetes Association guidelines. All patients underwent rest and adenosine stress myocardial perfusion CMR at a single mid-LV location, cine imaging covering the entire heart and myocardial tissue tagging in three LV short axis locations (apical, mid LV and basal). LV volumes and mass were calculated from cine images. LV rotation in three myocardial layers (endocardial, midmyocardial and epicardial) was calculated from CSPAMM tissue images using Tag Track (Gyrotools, Zurich). For this study, only rest MBF was calculated using Fermi-constrained deconvolution for the entire myocardium.

## Results

**Figure 1:**
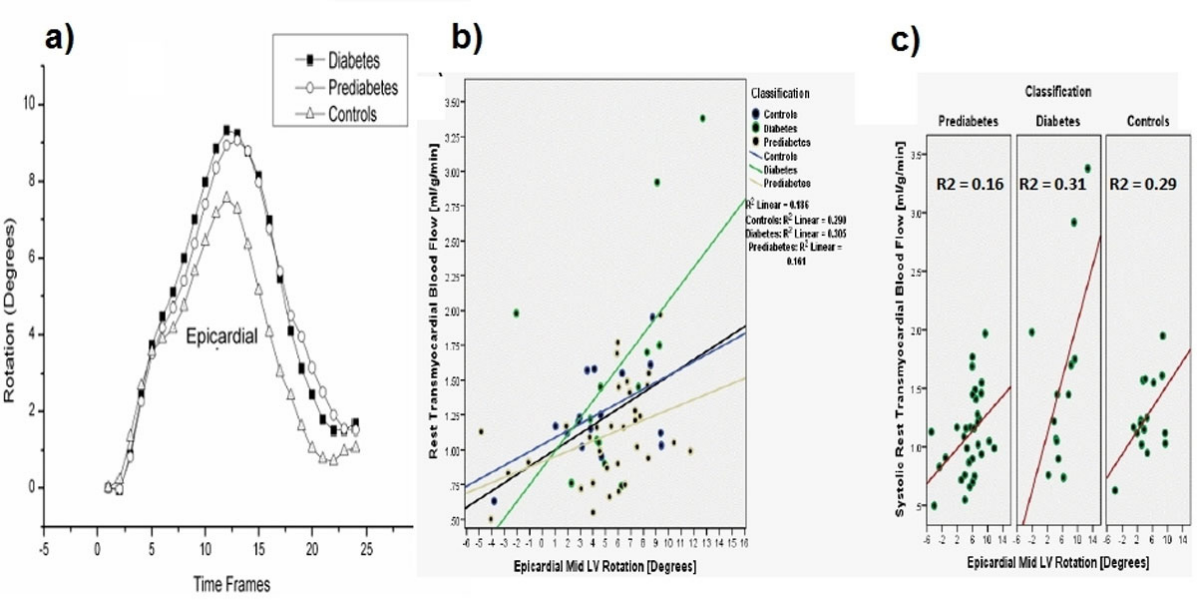
Figure 1 shows a mid slice LV epicardial rotation (a) and the linear determination coefficient (R2) between the response variable [resting MBF (ml/g/min), y axis] and the predictor variable [LV ER (Degrees) X axis] (b) and (c). The three patient individual groups are presented with colors.

Patient characteristics and results are shown in Table 1. Patients with T2DM had significantly higher resting MBF than the two other groups. Corresponding mid LV ER was higher in T2DM patients compared to patients with prediabetes and normal controls (Figure 1) (not statistically significant). Univariate analysis showed a significant positive correlation between MBF and LV ER in the entire study population (Pearson coefficient (r) of 0.25, p = 0.05). In the individual groups, the linear determination coefficient (R2) showed that LV ER in T2DM patients had stronger positive correlation (R2 = 0.31) with resting MBF than in patients with prediabetes (R2 = 0.16) and controls (R2 = 0.29), Figure 1.

**Table 1:** 

	T2DM	Prediabetes	Controls	P value
Number of patients :	n = 14	n = 34	n = 17	

Male, n / (%)	12 / (71)	15 / (44)	8 / (47)	n / a

Age, (years)	60 ± 6	56 ± 8	55 ± 7	ns

BMI, (kg/m^2^)	32 ± 5	30± 5	28 ± 4.4	0.04*

HbA1c, (mmol HbA1c / mol Hb)	64 ±16	41 ± 3	36 ± 2	

LV ejection fraction (%)	56 ± 6	59 ± 5	57 ± 5	ns

BSA Indexed LV mass (gram/m^2^)	54 ± 13	47 ± 7	50 ± 13	0.02*

Rest MBF, (ml/g/min)	0.83 ± 0.41	0.65 ± 0.24	0.78 ± 0.39	ns

Peak LV ER, (degrees) :				

Apical LV	10.05 ± 3.70	9.68 ± 3.44	7.82 ± 4.08	ns

Mid LV	5.9 ± 3.5	5.2 ± 3.7	4.3 ± 3.4	ns

Basal LV	2.41 ± 4.20	1.52 ± 2.90	0.55 ± 3.84	ns

BMI = Body Mass Index, BSA = Body Surface Area, n / a = not applicable, ns = not significant

## Conclusions

T2DM patients when compared to age-matched patients with prediabetes and normal controls have higher peak LV ER. Resting MBF correlates with LV ER. These results suggest that LV ER analysis in uncomplicated T2DM patients may be useful for non-invasive screening of early subtle contractile dysfunction associated with microvascular dysfunction in diabetes patients.

## Funding

This work has been funded by British heart foundation in part with the Libyan ministry for higher education and scientific research.

